# Vascular density and macular sensitivity in eyes after scleral buckling surgery for macula-on rhegmatogenous retinal detachment

**DOI:** 10.1371/journal.pone.0279683

**Published:** 2023-03-02

**Authors:** Przemyslaw Zabel, Katarzyna Zabel, Karolina Kazmierczak, Martyna Stankiewicz, Damian Jaworski, Karolina Suwala, Katarzyna Buszko, Joanna Stafiej, Grazyna Malukiewicz, Jakub J. Kaluzny

**Affiliations:** 1 Department of Sensory Organ Studies, Nicolaus Copernicus University, Collegium Medicum in Bydgoszcz, Bydgoszcz, Poland; 2 Department of Ophthalmology, Nicolaus Copernicus University, Collegium Medicum in Bydgoszcz, Bydgoszcz, Poland; 3 Oftalmika Eye Hospital, Bydgoszcz, Poland; 4 Division of Ophthalmology and Optometry, Department of Ophthalmology, Nicolaus Copernicus University, Collegium Medicum in Bydgoszcz, Bydgoszcz, Poland; 5 Department of Biostatistics and Biomedical Systems Theory, Nicolaus Copernicus University, Collegium Medicum in Bydgoszcz, Bydgoszcz, Poland; Yamagata University Faculty of Medicine: Yamagata Daigaku Igakubu Daigakuin Igakukei Kenkyuka, JAPAN

## Abstract

**Purpose:**

To investigate the structure and function of the retina after scleral buckling (SB) surgery due to macula-on rhegmatogenous retinal detachment (RRD).

**Methods:**

Twenty eyes with repaired macula-on RRD and 20 fellow eyes were included. All patients within 6–12 months of the procedure, were examined to evaluate retinal structure using spectral domain optical coherence tomography (SD-OCT) and vessel density (VD) by OCT angiography (OCTA). Best corrected visual acuity (BCVA) and microperimetry (MP) tests were used to assess retinal function.

**Results:**

Analysis of the microvascular network using OCTA between the operated and healthy fellow eyes showed a significant reduction on VD in superficial vascular plexus (SVP), deep vascular plexus (DVP) and radial peripapillary capillaries (RPC) (p< 0.001, p = 0.019 and p = 0.008, respectively). Comparison of retinal structure in SD-OCT showed no significant differences on thickness in ganglion cell complex (GCC) and peripaillary retinal nerve fiber layer (pRNFL) (p> 0.05) between examined eyes. Retinal function analysis by MP examination showed a decrease of retinal sensitivity (p = 0.0013) whereas postoperative BCVA showed no differences (p = 0.62) in the operated eyes. Significant Pearson’s correlations were observed between retinal sensitivity and VD in SVP, RPC (p< 0.05).

**Conclusion:**

In the eyes after SB surgery due to macula-on RRD, changes in retinal sensitivity were accompanied by impairment of the microvascular network assessed by the OCTA.

## Introduction

Retinal detachment, the separation of the neurosensory retina from the underlying retinal pigment epithelium, is a sight threatening condition that is considered one of the few ocular emergencies [1]. To maintain the best visual acuity, it is important to detect rhegmatogenous retinal detachment (RRD) as soon as possible in order to plan and prepare the patient for surgery. The options for surgical repair include pneumatic retinopexy, pars plana vitrectomy (PPV) and traditional scleral buckling (SB). While each of these techniques has advantages and limitations, the success rates are relatively high, so it is estimated that the first technique provides primary anatomical success in approximately 81% of the time, and two other surgery techniques in >90% of cases [2, 3]. Regarding the functional outcome, the main determinants include successful surgical reattachment of the retina and the status of the macula at presentation [4, 5]. Nevertheless, despite the fast surgical response, successful retinal reattachment, and lack of preoperative macular abnormalities seen on spectral domain optical coherence tomography (SD-OCT), some patients still report unsatisfactory postoperative vision [6, 7].

So far, visual acuity is the most frequently used method in clinical practice for assessing the result of postoperative visual function assessment, but it does not always allow for a complete and accurate assessment of the visual function. Patients who have been diagnosed with a macula-on before surgery usually have very good visual acuity after successful surgery, however, the postoperative quality of vision, stereopsis and near vision may not be fully satisfactory [8, 9]. The assessment of the retinal structure in the SD-OCT images most often indicates the normal architecture of the retina within the macula, which may raise questions as to whether and how macula-on RRD with retinal attachment in the posterior pole may affect macular function. Currently, we have more and more accurate techniques to study the function of the retina, such as microperimetry (MP), but also the use of OCT angiography (OCTA) can provide valuable information about the retinal vascular system using the flow characteristics.

Many studies have assessed treatment effects and the differences between SB and PPV regarding factors such as best corrected visual acuity (BCVA), retinal sensitivity measured using MP, reattachment rates, incidence of adverse events, SD-OCT findings and a few latest studies showed decreased retinal perfusion even after successful anatomical repair [10–13]. However, none of them analyzed the loss of function/structure as well as the density of the retinal vessels and did not correlate the obtained results in SD-OCT, OCTA and MP after SB in macula-on RRD. The hypothesis of our study was that in the eyes treated with a SB surgery for a primary RRD, despite preoperative attached macula and postoperative normal BCVA, the function and microvascular network of retina are disturbed, which can be thoroughly demonstrated by MP and OCTA.

The aim of the study was to analyze and compare the loss of retinal sensitivity, vessel density (VD), and the thickness of the inner retinal layers between eyes after successful SB surgery for macula-on RRD and the unaffected fellow eyes. Moreover, the correlations of the retinal function assessed by MP and BCVA tests with the structural changes of the retina obtained in the SD-OCT and OCTA studies were determined.

## Materials and methods

### Study design and patient recruitment

This was a cross-sectional, double-center study in which all patients underwent anatomically successful repair for primary macula-on RRD by SB surgery performed by retinal surgeons at the Department of Ophthalmology, Nicolaus Copernicus University, Bydgoszcz, Poland in the years 2020 to 2021. Postoperative ophthalmological examinations were carried out in Oftalmika Eye Hospital, Bydgoszcz, Poland. Participants signed written informed consent prior to their inclusion in the study following the tenets of the Declaration of Helsinki. The protocol of the study was approved by the Bioethical Commission of Nicolaus Copernicus University, Collegium Medicum in Bydgoszcz (KB 442/2020).

The collection of preoperative data included complete medical and ophthalmic history, measurement of BCVA, intraocular pressure (IOP), slit-lamp examination, and dilated fundus examinations. During qualification for surgery, special attention was placed on the status of the retina to exclude the presence of proliferative vitreoretinopathy, assess the macular status, clock-hours detached, number/location of breaks, and any possible media opacities including the lens status. Macula-on in RRD was confirmed in each case by the SD-OCT examination, which showed no separation of the neurosensory retina from the underlying retinal pigment epithelium in the area between the temporal arcades in the posterior pole of the eye.

Inclusion criteria were as follows: 1) successfully repaired primary RRD by a single, uncomplicated SB surgical procedure, 2) time from surgery to study inclusion between 6 and 14 months, and 3) macula-on without any macular involvement in the preoperative SD-OCT examination. Exclusion criteria were as follows: 1) preexisting pathological features of the macula or optic nerve, such as age-related macular degeneration, macular hole, vitreomacular traction syndrome, epiretinal membrane, intra- or subretinal fluid, glaucomatous neuropathy, 2) preoperative interocular refractive error >3.0 spherical equivalent (diopter), 3) amblyopia of any eye, 4) diabetes and a history of any retinal vascular occlusion, 5) a history of intraocular surgery, 6) visually significant cataract or other conditions that make it impossible to see the fundus of the eye, 7) traumatic RRD.

All subjects enrolled in this study underwent a detailed ophthalmological examinations within 6–14 months of the procedure, including the measurements of the following parameters: BCVA, slit-lamp biomicroscopy with Volk lens, IOP (Icare TAO1 i, Findland Oy, Vantaa, Finland), central corneal thickness (CCT) with an assessment of corneal endothelial cells density (CD) (Tomey EM-3000, Tomey Corporation, Japan), and axial length (AXL) measurement (IOL Master 500, Zeiss Humphrey, Dublin, CA, USA). The visual acuity tested on Snellen charts was converted into logarithm of the minimum angle of resolution (logMAR) for the purpose of statistical analysis. Additionally, these patients were examined SD-OCT (Heidelberg Engineering, Heidelberg, Germany), OCTA (Optovue, Inc., Fremont, CA, USA), and with the macular analyzer integrity assessment (MAIA) MP (Centervue, Padova, Italy). Participants were asked to refrain from consuming alcoholic and caffeinated beverages on the morning of the day of the study, as their consumption may affect blood pressure and interfere with OCTA results. Examinations were carried out over the course of one day, by the same ophthalmologist. All SD-OCT and OCTA examinations were assessed by an experienced retinal specialist (P.Z.).

**Fig 1:**
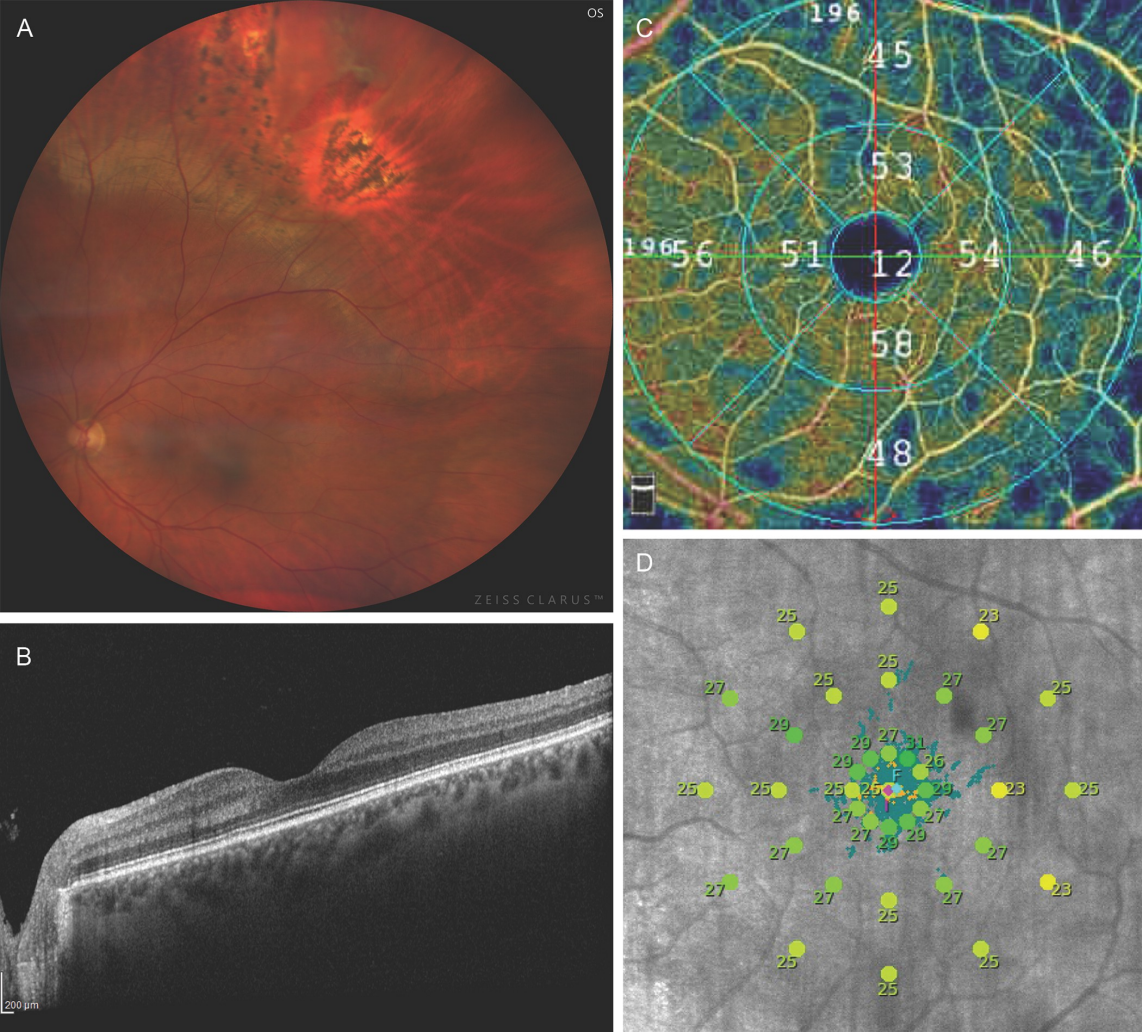
Left eye imaging results of a 57-year-old woman after scleral buckling for macula-on rhegmatogenous retinal detachment. A) Retinography showing the placement of the buckle, which was done such that the break would fall on the posterior 1/3 of the buckle; B) Spectral-domain optical coherence tomography demonstrating normal structure of the retina in the macula; C) Optical coherence tomography angiography presenting the vascular density in the superficial vascular plexus; D) Microperimetry result showing sensitivity of the retina around ± 5° of the macula.

All SB operations were performed in general anesthesia by two experienced surgeons (K.K. and J.S.). The procedure was carried out in the usual manner after preparation of the eye. Initially, conjunctival peritomy was made at 360, followed by rectus muscles were found and isolated with a silk suture. The eyeball was examined for abnormal vortex veins as well as for thinning of the sclera. A complete scleral depressed examination was performed. Next, in each quadrant were preplaced two mattress sutures of 6.0 MERSILENE® (Mersilk). In quadrants with a break, the anterior suture pass was usually performed at the level of muscle insertion with location adjustment to provide support to for the visualized tears, and the posterior suture pass was made 10 mm posteriorlyIn places with the band itself, the anterior and posterior suture passes were made about 12- and 16-mm posterior to the limbus. Attempts have been made to position the buckle so that the break would fall on the posterior 1/3 of the buckle. A circumferential segmental silicone tire (No. 287, MIRA, MIRA Inc., Waltham, MA) was placed only at the sites of the causative break(s), under the corresponding rectus muscle and the preinserted nonabsorbable sutures. A silicone encircling band (No. 240, MIRA, MIRA Inc., Waltham, MA) was besteaded through the groove of the tire and circumferentially 360° (in preinserted sutures and under the rectus muscles). If necessary, external subretinal fluid drainage was performed at a site beneath the buckle. Cryotherapy was applied on the retinal break(s). The band and tire were attached to the sclera by tying the preplaced sutures. Then two ends of the band were passed through a silicone muff. Indirect ophthalmoscopy was done to confirm appropriate support of the break with adjustment of the buckle until it achieved the desired height after which two ends of the band in the muff was tightened. The excess ends of the band were cut 2–3mm away from the muff. The conjunctiva was sutured with 6.0 Vicryl suture (Fig 1).

### Spectral domain optical coherence tomography

SD-OCT imaging was performed to assess macular structure and to measure peripapillary retinal nerve fiber layer (pRNFL) and ganglion cell complex (GCC) thickness using two devices. The SD-OCT, Spectralis (Heidelberg Engineering, Heidelberg, Germany) was used to study the macula and peripapillary area. Macular structure assessment was conducted using the posterior pole ‘p. pole’ protocol which encompasses a 30×25° scan with 61 scans per section. We evaluated the structure of the retina to exclude the presence of vitreoretinal interface abnormalities and intra- or subretinal fluid for each scan. Using the glaucoma protocol, global thickness of the pRNFL was analyzed over 360° within a circular scan that consisted of 768 A-scans. The scanned circle was 3.46 mm in diameter and was concentered with the optic nerve head (ONH). To measure the thickness of the retinal GCC, the built-in Avanti RTVue XR (Optovue, Inc., Fremont, CA, USA) glaucoma module was used. The GCC scan was centered 1 mm temporal from the fovea and covered a circular macula area of 6 mm in diameter.

### Optical coherence tomography angiography

OCTA imaging was carried out with an Avanti RTVue XR (Optovue, Inc., Fremont, CA, USA) device with AngioVue software (version 2017.1.0.151), which provides non-invasive qualitative visualization and quantitative assessment of the retinal vascular network using the split-spectrum amplitude-decorrelation angiography algorithm. To correct motion artifacts, OCTA combines orthogonal fast-scan directions (horizontal and vertical) and is equipped with DualTrac Motion Correction Technology. The software we use is equipped with three-dimensional Projection Artifact Removal; therefore, projection artifacts have been reduced in all deeper retinal layers while maintaining their authentic layout. The macula was analyzed by using B-scans covering an area of 6 × 6 mm^2^ while on the area of 4.5 × 4.5 mm^2^ centered on the ONH, the peripapillary vessels were analyzed. The images consisted of two sets of B-scans repeated horizontally and vertically, each consisting of 400 A-scans.

The data were analyzed with commercially available software consisting of automatic segmentation of the superficial vascular plexus (SVP) and deep vascular plexus (DVP) of the macula and the peripapillary radial peripapillary capillary (RPC) layer in the ONH area, and then automatic measurement of the vessel density in these plexuses as well as the analysis of the surface area in the foveal avascular zone in the macular area. VD was calculated as the percentage of area occupied by flowing blood vessels in the selected region. For ONH scans, VD was analyzed in the peripapillary area, which extends outwards from the ONH border with an elliptical area between 2–4 mm. The RPC layer was defined as extending from the inner limiting membrane (ILM) to the posterior border of the RNFL. In the macula, analyses of vessel density were performed on the entire surface of 6 × 6 mm^2^ en face images. The SVP comprised the area between the ILM and the outer boundary of the inner plexiform layer (IPL), while the DVP comprised the area between the outer boundary of the IPL and the outer boundary of the outer plexiform layer. Only measurements of good technical quality with a scan quality (SQ) of 6 or more on a 10-degree scale, with which a commercial camera is equipped, qualified for further analysis. Measurements with motion artifacts present on the en face images (irregular patterns of vessels or a blurred boundary of the ONH) were also rejected.

### Microperimetry

MP MAIA (Centervue, Padova, Italy) combines scanning laser ophthalmoscopy, static perimetry, fixation analysis and fundus imaging. The mechanism of observation is an infrared superluminescent diode of wavelength 850 nm, which provides images of high quality even with pupil diameter of 2.5 mm. Maximum level of illumination is 318.47 cd /m2. This light is distributed in attenuation from 0 to 36 dB in steps of 1 dB. The background luminance is 1.27 cd / m2. The stimulus is predetermined to be size III Goldmann type and each one is presented during a period of 200 ms. MP delivers information in the form of retinal threshold sensitivity and fixation stability. Average threshold (AT) of retinal sensitivity was measured with the expert exam option, using the MAIA standard macular grid pattern (37 stimuli points) over 10 retinal degrees (±5° around the macula). The projection strategy was the standard 4–2 and all measurements were done prior to administering the mydriatics, in a quiet, dark room, and the lights were turned off after applying the veil to the untested eye. Patients were instructed to look at the center of the fixation target during the examination. Eye movements were registered during examination by an integrated eye-tracker system with a frequency of 25 Hz, with the double purpose of providing correction of ocular misalignments and registration of subjects’ fixation pattern during the exam.

### Statistical analysis

Descriptive statistics for normally distributed data were presented as a mean ± standard deviation (SD). In case of categorical variables, the percentage structure indexes were applied. Normal distribution of the data was assessed by the Shapiro-Wilk test. Depending on the results of the test, the differences between continuous variables were analyzed using t-Student’s test or the Wilcoxon’s tests. The Pearson’s correlation coefficients were determined in order to assess the linear associations between the continuous variables, the tests for significance of correlation coefficients were also performed. The LMM model was applied to analyze the association between SVP, DVP, RPC and eyes (operated, fellow) and SQ as independent variables. All statistical analyzes were conducted at significance level *α* = 0.05. All statistical calculations were performed in the R software (version 3.6.2) and Statistica (version 13.2).

## Results

Initially, twenty-six patients agreed to participate in the study. Six of them were excluded because of movement artifacts or media opacities affecting to the low of SQ. Finally, 20 eyes with successfully repaired macula-on RRD and 20 fellow healthy eyes of twenty patients (11 female and 9 male) were included to the study. The mean patient age was 58.1 ± 11.46 years. The average time between surgery and examination time was 9.15 ± 2.79 months and the duration of symptoms was 4.8 ± 2.42 days. All enrolled participants had their own clear lenses in both eyes. The average number of retinal breaks was 1.3 ± 0.57. Most prevalent eyes had an RRD extending 1 quadrant (12 of 20 eyes, 60%), followed by 2 quadrants (8 of 20 eyes, 40%). Detachment was located in the superior hemisphere of the retina in 18 (80%) eyes, inferior hemisphere in 2 (10%) eyes, and involved both superior and inferior hemispheres also in 2 (10%) eyes. Demographic and clinical characteristics are presented in Table 1.

**Table 1:** Demographic and clinical data of patients.

Variables	Description
Number of patients/operated eyes (n, %)	n = 20
Right eyes	14 (70)
Left eyes	6 (30)
Sex (n, %)	
Female	11 (55)
Male	9 (45)
Age (years, mean ± SD)	58.1±11.46
Duration of symptoms (days, mean ± SD)	4.8±2.42
Time between surgery and examination (months, mean ± SD)	9.15 ±2.79
Number of breaks (mean ± SD)	1.30 ± 0.57
Extent of retinal detachment (n, %)	
1 quadrant	12 (60)
2 quadrants	8 (40)
Detachment location (n, %)	
Superior hemisphere	16 (80)
Inferior hemisphere	2 (10)
Both hemispheres	2 (10)

The eyes after SB showed no significant differences in IOP, CCT, endothelial CD, and AXL compared to fellow eyes (p> 0.05). Preoperative and after SB surgery, BCVA was slightly worse in the eyes with macula-on RRD, but the differences in both cases were not statistically significant (p = 0.29 and p = 0.62, respectively). The analysis of the retinal sensitivity by MAIA MP showed a significant decrease of AT in the eyes subjected to surgery compared to the fellow eyes (p = 0.0013). The functional and morphological results of the eyes enrolled in the study are presented in Table 2.

**Table 2:** Comparison of functional and morphological characteristics between fellow eyes.

Parameter	RRD Eyes	Fellow Eyes	P-value
AT (dB, mean ± SD)	24.37 ± 1.93	26.09 ± 0.94	0.0013
Preoperative BCVA (logMAR, mean ± SD)	0.08 ± 0.12	0.04 ± 0.09	0.29
Postoperative BCVA (logMAR, mean ± SD)	0.05 ± 0.08	0.04 ± 0.09	0.62
IOP (mmHg, mean ± SD)	17.93 ± 3.17	18.94 ± 3.14	0.57
CCT (μm, mean ± SD)	526.69 ± 39.34	529.25 ± 41.45	0.86
CD (cell/mm^2^, mean ± SD)	2261 ± 374.53	2414.56 ± 279.81	0.21
AXL (mm, mean ± SD)	25.31 ± 1.35	24.85 ± 1.29	0.29

Statistical significance tested by t-Student’s test or the Wilcoxon’s tests.

P-value <0.05 was considered to be statistically significant.

Abbreviations: RRD, rhegmatogenous retinal detachment; AT, average threshold; BCVA, best corrected visual acuity; IOP, intraocular pressure; CCT, central corneal thickness; CD, cell density; AXL, axial length.

**Fig 2:**
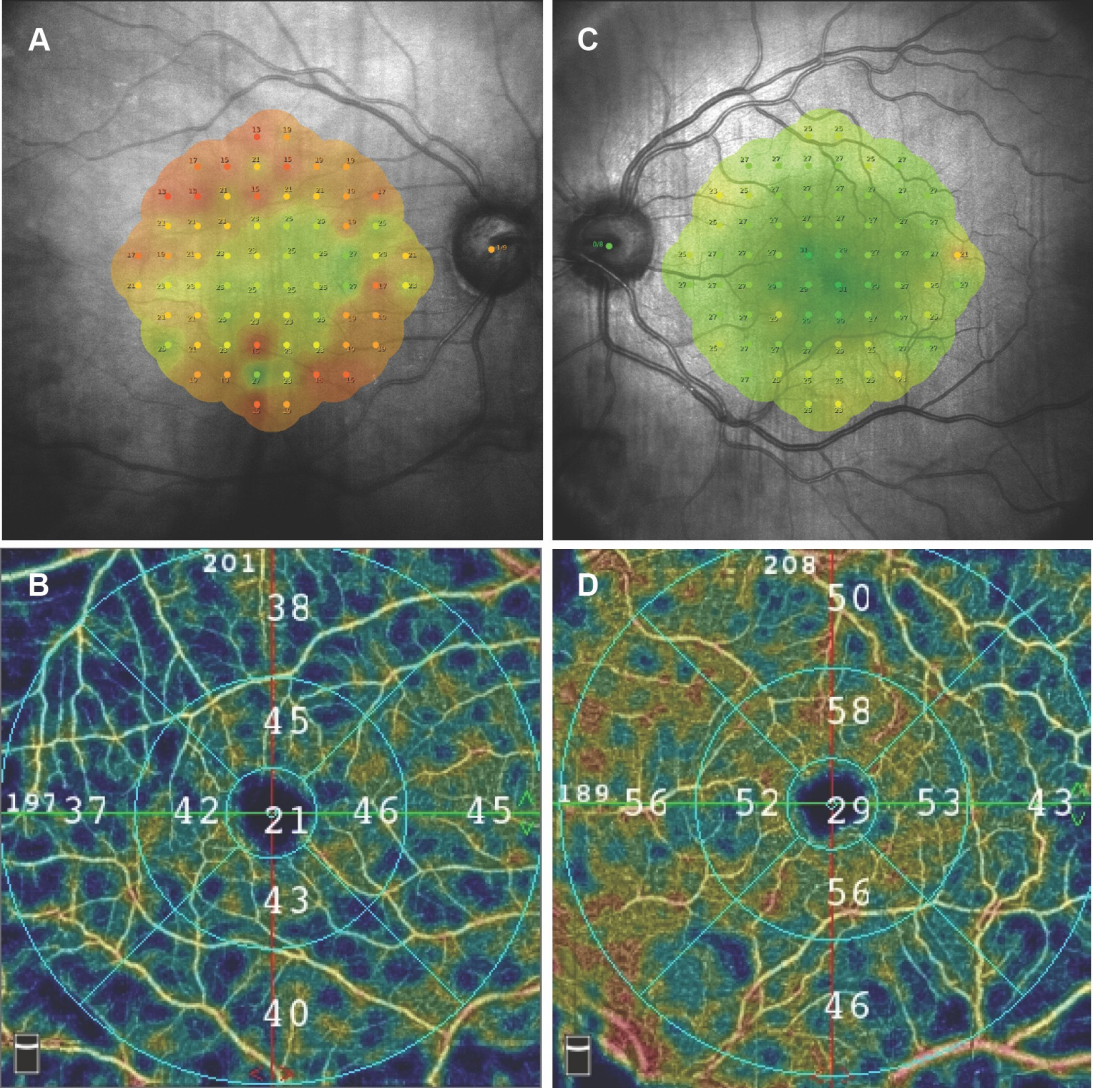
The case of a 52-year-old female patient after scleral buckling surgery for macula-on rhegmatogenous retinal detachment of the right eye. A) Decreased retinal sensitivity threshold in the microperimetric examination of the operated eye; B) Reduced vessel density in en face optical coherence tomography angiogram of superficial retinal vascular plexus in the operated eye; C) Retinal sensitivity threshold in the microperimetric examination of the healthy fellow eye within normal limits; D) Vessel density in en face optical coherence tomography angiogram of superficial retinal vascular plexus in the healthy fellow eye within normal limits.

In the macula, analysis of microvascular network using OCTA between the operated and fellow eyes on the entire surface of 6x6 mm^2^ en face images showed a significant reduction on VD in SVP and DVP (p< 0.001 and p = 0.019, respectively) (Fig 2). In the foveal area of the Early Treatment of Diabetic Retinopathy Study chart (i.e., inner 1-mm diameter circle) no significant differences were founds on VD in any plexus (p> 0.05), as opposed to the parafoveal area (rings between 1 mm and 3 mm from the center of the fovea) and perifoveal area (rings between 3 mm and 6 mm from the center of the fovea) where these differences were significant (p< 0.05). The reduction on VD in the eyes after SB was also significant in the RPC layer (p = 0.008). There were no differences between the eyes in the foveal avascular zone area (p = 0.71). Additionally, the comparative analysis of the structure of the retina between the eye after SB and the fellow eyes showed no significant differences on thickness in both GCC and pRNFL (p = 0.24 and p = 0.71, respectively). The microvascular network and structural results of the eyes enrolled in the study are presented in Table 3.

**Table 3:** Comparison of microvascular network and structural characteristics between fellow eyes.

Parameter	RRD Eyes	Fellow Eyes	P-value
Mean VD (%, mean ± SD)			
SVP	44.54 ± 4.02	49.36 ± 3.04	<0.001
DVP	43.77 ± 4.17	48.07 ± 5.44	0.019
Fovea VD (%, mean ± SD)			
SVP	22.76 ± 8.14	23.17 ± 8.84	0.88
DVP	36.97 ± 8.05	39.04 ± 6.51	0.38
Parafovea VD (%, mean ± SD)			
SVP	46.87 ± 5.26	52.43 ± 3.11	<0.001
DVP	50.52 ± 3.58	53.59 ± 3.51	0.009
Perifovea VD (%, mean ± SD)			
SVP	45.36 ± 4.07	49.75 ± 2.97	<0.001
DVP	43.78 ± 5.07	50.03 ± 5.38	<0.001
RPC VD (%, mean ± SD)	47.92 ± 5.19	51.08 ± 2.21	0.008
FAZ (mm^2^, mean ± SD)	0.257 ± 0.102	0.245 ± 0.94	0.71
GCC (μm, mean ± SD)	93.65 ± 9.95	97.05 ± 8.07	0.24
pRNFL (μm, mean ± SD)	94.58 ± 12.58	95.95 ± 10.01	0.71
OCTA SQ index (mean ± SD)	7.31 ± 0.81	7.65 ± 0.81	0.18

Statistical significance tested by t-Student’s test or the Wilcoxon’s tests.

P-values adjusted for SQ (in SVP, DVP and RPC), based on Linear Mixed Effects Model.

P-value <0.05 was considered to be statistically significant.

Abbreviations: RRD, rhegmatogenous retinal detachment; VD, vessel density; SVP, superficial vascular plexus; DVP, deep vascular plexus; RPC, radial peripapillary capillaries; FAZ, Foveal avascular zone; GCC, ganglion cell complex; pRNFL, peripapillary retinal nerve fiber layer; OCTA, optical coherence tomography; SQ, scan quality.

**Fig 3:**
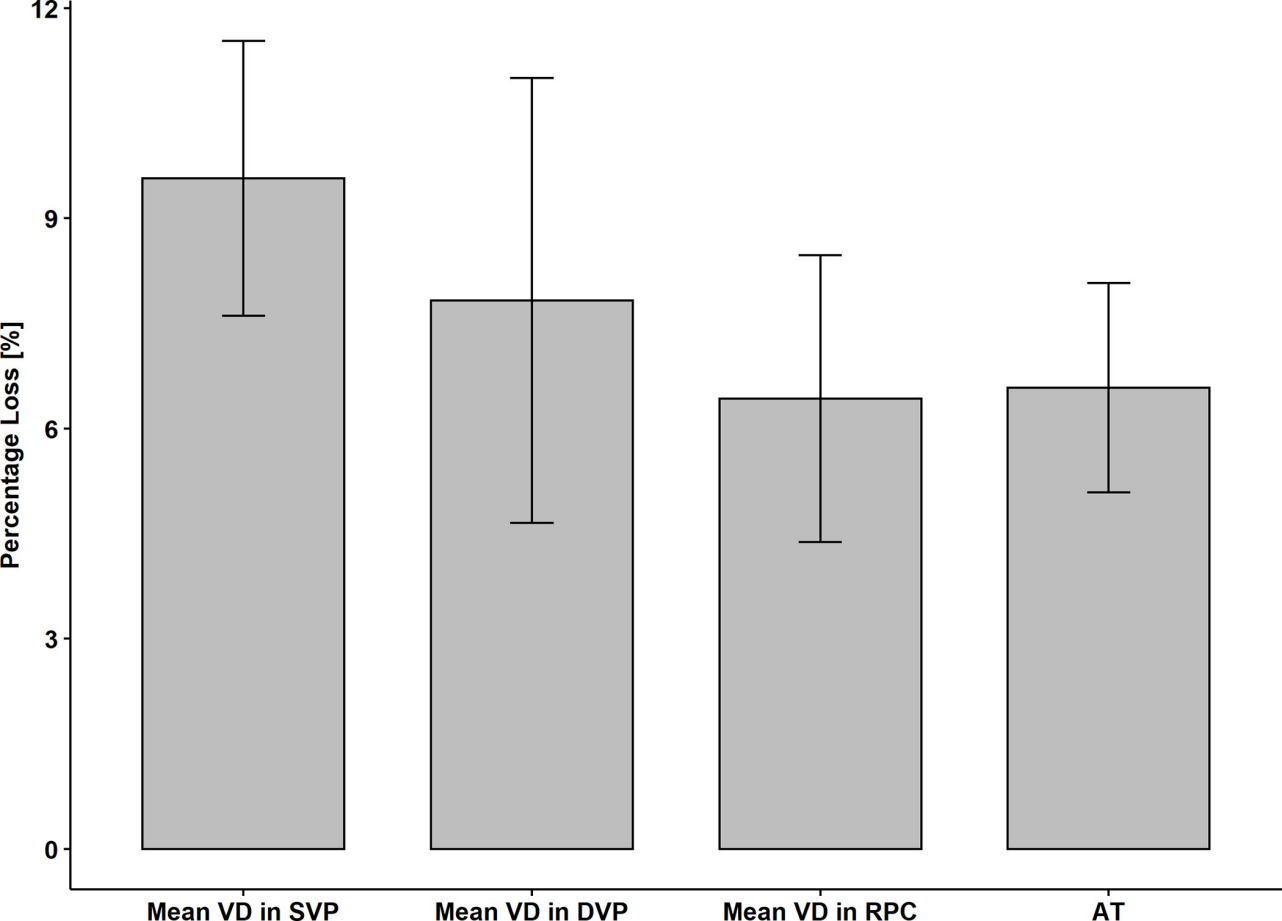
The box plot presents a direct comparison of the percentage loss of vessel density and average threshold of the retinal sensitivity after scleral buckling surgery for macula-on retinal detachment.

The percentage loss in the AT and the microvascular network of the retina after SB surgery, which showed significant statistical differences between the operated and fellow eyes, are shown in Fig 2. The greatest percentage loss was shown on VD in SVP (9.57 ± 8.76), then on VD in DVP (7.83 ± 14.19), AT of the retina in MP (6.58 ± 6.68) and VD in RPC (6.43 ± 9.16). The differences between these parameters did not show statistical significance (p> 0.05) (Fig 3).

**Fig 4:**
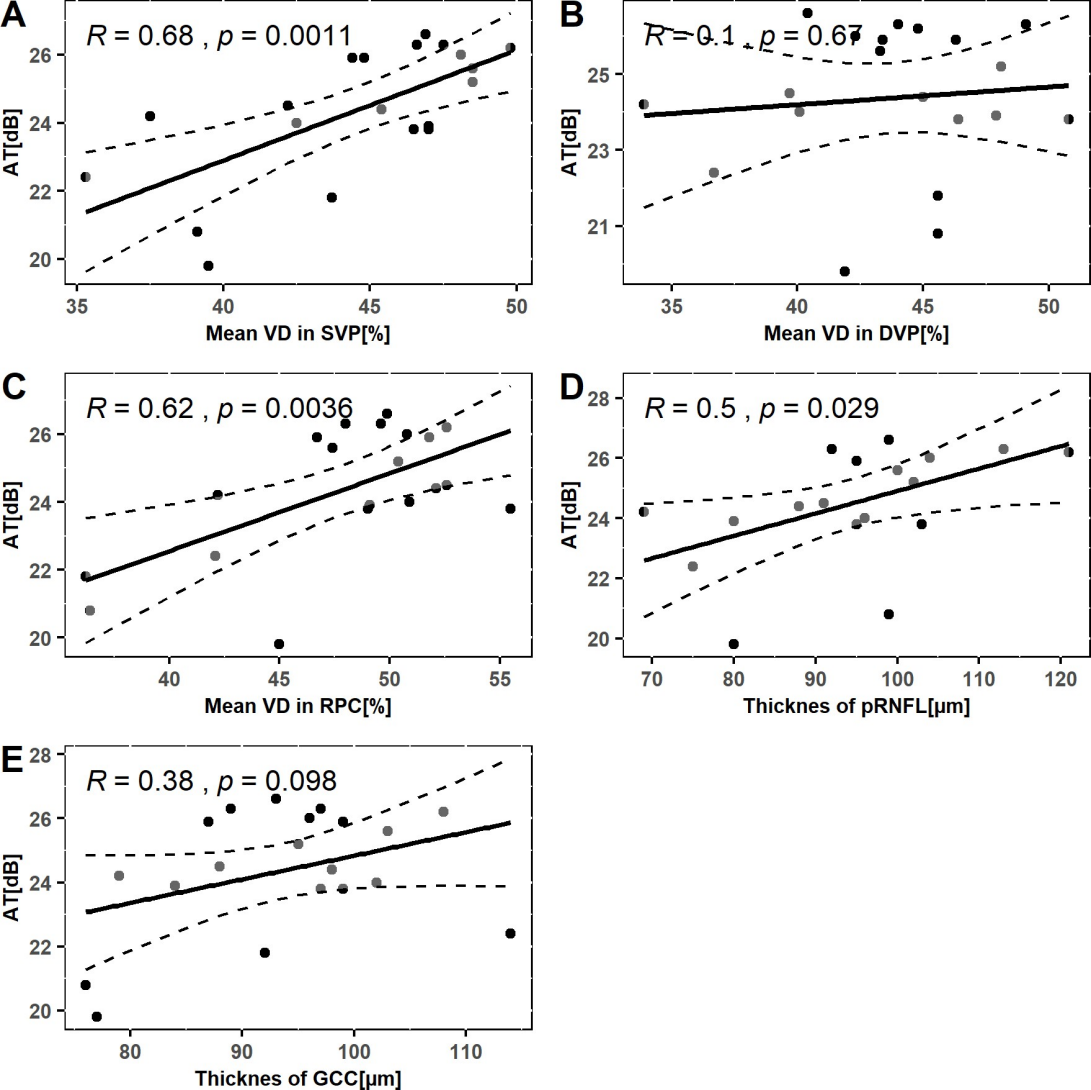
The scatter plots show the Pearson’s correlation coefficient measuring the relationship between. A) average threshold of the retinal sensitivity and mean vessel density in superficial vascular plexus; B) average threshold of the retinal sensitivity and mean vessel density in deep vascular plexus; C) average threshold of the retinal sensitivity and mean vessel density in radial peripapillary capillaries; E) average threshold of the retinal sensitivity and thickness of peripapillary retinal nerve fiber layer; F) average threshold of the retinal sensitivity and thickness of ganglion cell complex; of eyes after scleral buckling surgery for macula-on retinal detachment.

**Fig 5:**
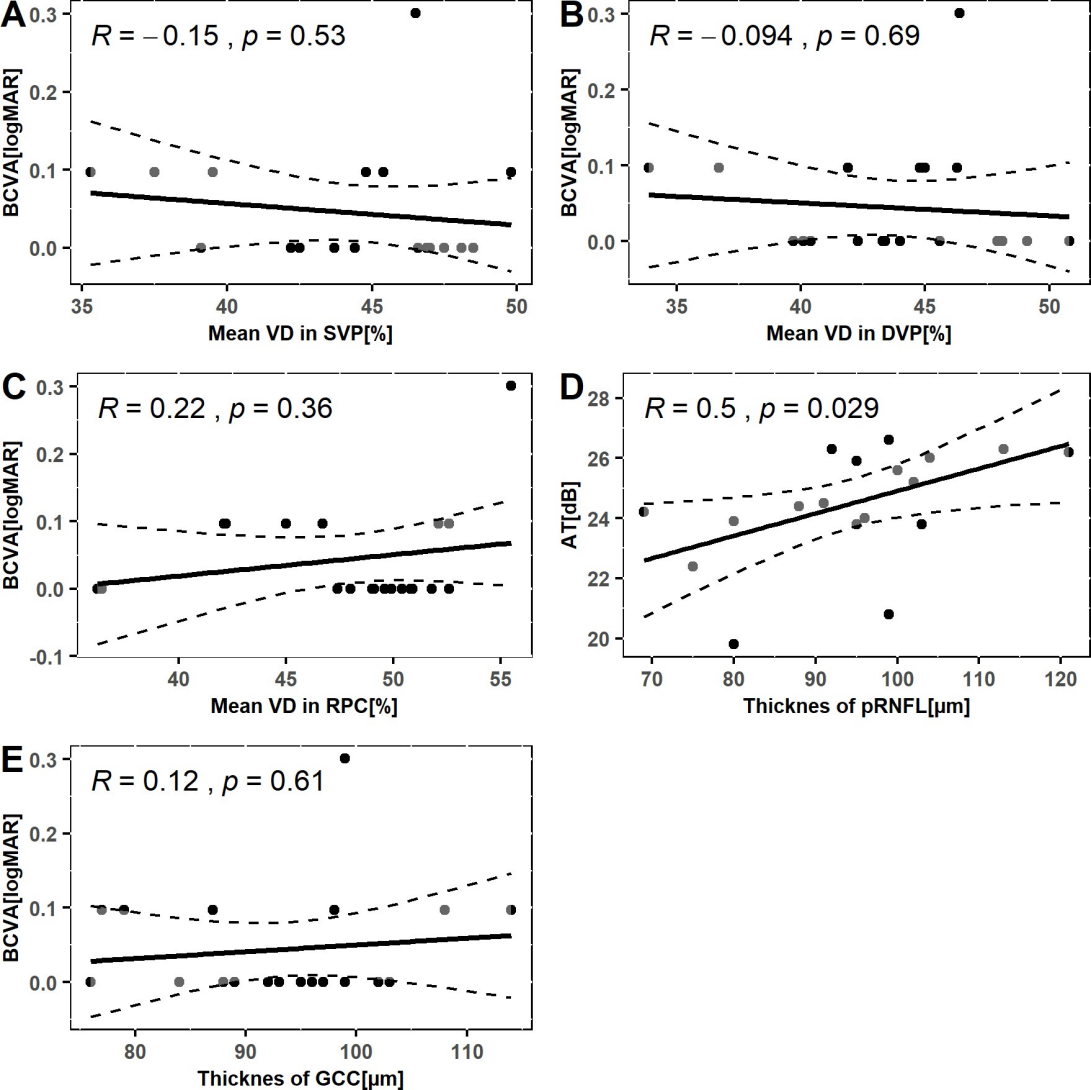
The scatter plots show the Pearson’s correlation coefficient measuring the relationship between. A) best corrected visual acuity and mean vessel density in superficial vascular plexus; B) best corrected visual acuity and mean vessel density in deep vascular plexus; C) best corrected visual acuity and mean vessel density in radial peripapillary capillaries; D) best corrected visual acuity and thickness of peripapillary retinal nerve fiber layer; E) best corrected visual acuity and thickness of ganglion cell complex; of eyes after scleral buckling surgery for macula-on retinal detachment.

The scatter plots shown in Figs 4 and 5 illustrate the relationship between functional parameters of the retina and structural parameters in the macular and peripapillary areas. Significant Pearson’s correlations were observed between retinal AT and VD in SVP, RPC, and pRNFL thickness. The strongest correlation was found between AT and mean VD in SVP (Pearson’s R = 0.68, p = 0.0011). Functional assessment of the retina by postoperative BCVA showed no relationship to any structural parameter (Figs 4 and 5).

## Discussion

To our knowledge, this is the first study which compares the results of retinal function and structure using MP and SD-OCT as well as the microvascular network imaged with OCTA between eyes treated with a SB surgery for a macula-on RRD and fellow eyes.

This study found that retinal detachment caused the postoperative retinal AT to be significantly lower than in the fellow eyes, even when preoperative RRD did not involve the macula. The second parameter that is more commonly used to assess retinal function is BCVA, but it showed no difference between eyes after surgery for macular-on RRD without postoperative complications and abnormal SD-OCT findings, and fellow eyes. Previous reports also confirm that visual acuity evaluation may be a poor predictor of many aspects of their visual functions [9, 14, 15]. BCVA is a parameter that subjectively and not very precisely determines the function of the retina after it is reattached, and it is known that it depends most on the involvement of the macula. Our results confirms that when the macula was not detached, other, more sensitive methods of assessing macular function are needed, because even with normal BCVA, AT of retinal sensitivity may be reduced. Although we did not find reports using the MP test to assess and compare the function of the retina after SB due to macula-on RRD, our results were consistent with similar, previous studies comparing retinal function between eyes with macula-on RRD and fellow eyes. Okamoto et al. assessed the changes in contrast sensitivity after unilateral macula-on RRD undergoing SB or PPV. They showed that the contrast sensitivity decreased significantly after surgery, but logMAR BCVA did not change [16]. In 2019, Akiyama et al. performed focal macular electroretinography (FMERG) to assess macular function in patients with macula-on RRD. They found that the amplitudes of a- and b-waves and oscillatory potentials were significantly lower for eyes with macula-on RRD than in fellow eyes. Although the altered FMERG responses suggested the presence of macular dysfunction in eyes with macula-on RRD, the mechanism underlying these results is still unconfirmed [17]. There are suspicions that retinal ischemia at the detached areas in eyes with macula-on RRD reduces blood flow within the macula and ONH [17, 18].

Previous studies in this field using fluorescein angiography have shown a decrease in retinal blood flow in patients with RRD, which was caused by an increase of the peripheral resistances [19]. Another report using scanning laser Doppler flowmetry, also showed a reduced flow in the macular microcirculation in patients with primary RRD without macular involvement. The flow reduction in the retinal microcirculation was dependent on the extent of RRD [20]. Roldán-Pallarés et al. analyzed the preoperative hemodynamic parameters of the central retinal artery (CRA) using a color-Doppler ultrasound. They showed lower CRA flow parameters in RRD eyes, which was related to the preoperative duration of RRD [21]. However, this research on retinal vascularization did not analyze quantitative parameters of microvasculature in different retinal plexuses. The use of OCTA makes it possible to separately assess the microvascular network in a qualitative and quantitative manner at different depths of the retina, distinguishing individual plexuses that may be involved in various pathological processes.

In our research, OCTA imaging was used to evaluate individual retinal plexuses that we correlated with functional parameters. Bonfiglio et al. in the eyes after PPV due to macula-on RRD, found a lower VD in OCTA images only in parafoveal DVP, which was related postoperatively to BCVA [22]. Contrary to their results, we showed a decrease of VD in SVP and RPC as well as in DVP. The reason for the difference in the obtained results may be the method of surgery, as our patients underwent SB. The analysis of correlations between VD in OCTA and functional parameters of the retina showed no relationship with postoperative BCVA but with the AT of the retinal sensitivity in the MP examination. This confirms the suspicion that that BCVA is not a precise parameter, especially if the macular area has not been detached. In turn, Barca et al. assessed retinal vascularization in OCTA in patients undergoing RRD surgery repair, including SB and PPV. They showed that in the first months after surgery, VD in whole SVP in the affected eyes was significantly lower than that of the fellow eyes; nevertheless, this difference between the two groups disappeared at 6 months after surgery. However, there were no significant differences in VD over the entire follow-up period in DVP after surgery compared with fellow eyes. Moreover, they demonstrated that VD of SVP in the affected eye, despite the lack of involvement of the macula, correlated with preoperative BCVA. Unfortunately, they did not separate the eyes in which SB or PPV surgery was performed for statistical analyzes, which could have an impact on the obtained results [23]. Contrary to their study, where after 6 months of follow-up there were no differences on VD in both SVP and DVP, our results confirmed a sustained reduction on VD in SVP, DVP and RPC. Moreover, the greatest percentage losses in the microvascular network were related to SVP.

In primary RRD, and even more so without macular involvement, SVP appears to be the most-affected plexus because it may be the first vascular layer involved in the rapid increase in vascular resistance induced by RRD. This stronger and faster contraction in SVP compared to DVP which is observed in OCTA in the form of a decrease VD may occur due to the greater density in arterioles and in smooth muscle. Based on knowledge of chronic pathologies such as diabetic retinopathy, where the purported pathogens are slower than those in primary RRD, it can be concluded that DVP, even if it is more susceptible to hypoxic damage, may be involved later. Because DVP reportedly contributes 10% to 15% of the oxygen supply to the photoreceptors, reduced VD in the DVP may be associated with an outer retinal change and impaired retinal function [24]. Scarinci et al. reported that areas of capillary nonperfusion in DVP due to macular ischemia are associated with photoreceptor structural abnormalities, compromised VA and retinal sensitivity loss on MP [25, 26]. We suspect that also the type of surgery may have an impact on the OCTA results due to the observed VD changes in SVP, DVP and RPC. This may also be confirmed by reports of iris perfusion occurring after SB surgery for RRD. D’Aloisio et al. demonstrated that the SB surgical technique is associated with a significant reduction in overall iris perfusion as demonstrated by OCTA. The reduction in perfusion may be secondary to the mechanical stress on the vessels that also supply the iris. In addition, tension of the ocular rectus muscles and the application of a 360° encirclement might cause serious hypoperfusion [27].

Other important proven factors involved in VD reduction are the release of endothelin-1 as well as the role of Müller cells in the pathological process. Increase in the level of endothelin-1, a vasoconstrictor peptide in the subretinal fluid, the action of which causes the narrowing of the retinal blood vessels and, consequently, a reduction in blood flow [28, 29]. Endothelin-1 also acts on retinal Müller cells, promoting their activation throughout the retina [30, 31]. Activation of Müller cells may affect the retinal vascularization in the form of cell hyperplasia, gliosis and subretinal fibrosis, in the absence of macrostructural changes [32, 33]. This abnormal mechanism may explain to some extent how the macular blood flow is reduced, even though the detachment was outside the macula.

Our study has several limitations. First, the sample size was relatively small because this study focused on primary macula-on RRD and excluded patients with macular detachment, media opacities, which limited the number of selected patients. For this reason, we included patients with different follow-up periods that were previously defined in the inclusion criteria. Second, the OCTA examination was performed after surgery, so we do not know the preoperative microcirculation status and we cannot determine whether the macular vascular impairment is caused by RRD or surgery. Furthermore, the retrospective design precludes the assessment of changes over time, therefore a future multicentre prospective study based on this preliminary work would provide significant anatomical and functional parameters for visual changes over time.

In conclusion, we found that there was no significant change in BCVA after SB surgery for primary RRD with macula-on, but detailed evaluation of retinal function by MP revealed a decrease in retinal AT sensitivity in comparison with the fellow eye. The changes in retinal AT sensitivity were accompanied by impairment to the microvascular network, especially in SVP, but also in RPC and DVP assessed by the OCTA. However, the advantage of any of the mechanisms in the form of released proteins or the performed surgery affecting the retinal AT sensitivity and microvasculature network is uncertain, therefore it is worth conducting appropriate further studies to assess their impact.

## Supporting information

S1 Data (XLSX)
